# Special Issue on “The Tight Junction and Its Proteins: More than Just a Barrier”

**DOI:** 10.3390/ijms21134612

**Published:** 2020-06-29

**Authors:** Susanne M. Krug, Michael Fromm

**Affiliations:** Institute of Clinical Physiology/Nutritional Medicine, Department of Gastroenterology, Rheumatology and Infectious Diseases, Charité—Universitätsmedizin Berlin, Campus Benjamin Franklin, 12203 Berlin, Germany; susanne.m.krug@charite.de

**Keywords:** tight junction, claudin, occludin, tricellulin, angulin, paracellular channels and barriers, cell polarity, pathogens, immune cells, environmental sensors, cancer

## Abstract

For a long time, the tight junction (TJ) was known to form and regulate the paracellular barrier between epithelia and endothelial cell sheets. Starting shortly after the discovery of the proteins forming the TJ—mainly, the two families of claudins and TAMPs—several other functions have been discovered, a striking one being the surprising finding that some claudins form paracellular channels for small ions and/or water. This Special Issue covers numerous dedicated topics including pathogens affecting the TJ barrier, TJ regulation via immune cells, the TJ as a therapeutic target, TJ and cell polarity, the function of and regulation by proteins of the tricellular TJ, the TJ as a regulator of cellular processes, organ- and tissue-specific functions, TJs as sensors and reactors to environmental conditions, and last, but not least, TJ proteins and cancer. It is not surprising that due to this diversity of topics and functions, the still-young field of TJ research is growing fast. This Editorial gives an introduction to all 43 papers of the Special Issue in a structured topical order.

## 1. Introduction

The aim of the Special Issue “The Tight Junction and Its Proteins: More Than Just a Barrier” is to collect original studies and reviews on the functions of the tight junction (TJ) and its proteins, which extend beyond the classical, and therefore name-giving, task of sealing the paracellular pathway of epithelia. Terms like “tight” junction, occludin and claudin (both derived from Latin words indicating closure) are suggestive of a sole barrier function for these structures. However, there are many other functions, including those in gene expression, morphogenesis, cell proliferation, adhesion, signaling, immune responses, reaction to bacteria and viruses, cancer, and the enhancement of drug uptake. All 43 articles of this Special Issue are briefly introduced in Section 2 to Section 6 of this editorial review.

## 2. Tight Junction Barriers and Structures

As briefly delineated by the first section, the TJ is mainly known as being structure forming and regulating the paracellular barrier. However, besides the dynamic and complex regulation of the paracellular pathways, many other aspects have been discovered where TJs and their proteins are involved, indicating that there are many functions beyond this.

In this Special Issue, we would like to introduce and cover several of the new arising functions. However, even the classical function as a paracellular transport-regulating protein complex is still not completely understood, and regulation in health and disease, as well as the molecular and structural aspects of TJs and their components, need to be fully elucidated.

In the following sections, a short overview of the articles within this Special Issue is given to indicate the different facets of TJ protein functions that may extend beyond the typical paracellular barrier aspects.

### 2.1. Pathogens Affecting the TJ and Regulation via Immune Cells 

The barrier function of the TJ helps to protect the organisms against the entry of pathogens from the outer environment and thus is often an initial target of pathogens, leading to barrier impairment and subsequent inflammation processes in disease statuses. Many studies have shown direct interactions of pathogens with TJ proteins or indirect influence due to their secreted toxins aiming to break down the barrier. The impairment of the intestinal barrier leads to osmotic imbalances and diarrhea as the main symptoms and can be caused by many pathogenic bacteria. However, many pathogenic bacteria do not only act on TJs directly but also induce apoptosis and thereby the loss of epithelial integrity. For example, *Klebsiella oxytoca*-induced antibiotic-associated hemorrhagic colitis and diarrhea occur mainly to the secreted cytotoxins tilivalline and tilimycin, which induce apoptosis, while in addition, independently of these, *Klebsiella oxytoca* also affects claudin-5 and -8 and thus paracellular barrier integrity [1]. Toxins like *Clostridium perfringens* enterotoxins (CPEs) are also known to directly interact with claudins and have come into focus for use as tools targeting claudins specifically [2,3].

Inflammatory processes, which often go together with barrier impairment, are linked to activated immune cells reacting upon enhanced antigen uptake or other stimuli. Cytokines that are secreted not only further stimulate immune cells but also change the regulation and expression of TJ proteins, affecting barrier properties. In airway epithelia, the secretion of interleukin-13 (IL-13) induces the ubiquitination of TJ proteins and leads to proteasomal aggregation via the JAK/STAT-dependent expression of ubiquitin-conjugating E2 enzyme, which finally results in barrier disruption and lung damage [4]. Immune cells, furthermore, usually balance barrier-disturbing and -protective cytokine secretion; for example, IL-4 inhibits the barrier-enhancing effects of IL-17A, and an imbalance of such cytokine profiles has been shown in atopic dermatitis [5]. Furthermore, in other pathologies, changes in the cytokine patterns of immune cells seem to be key factors for barrier defects. In celiac disease, peripheral monocytes possess elevated pro-inflammatory cytokine levels, altering the TJ’s protein composition and thus the barrier function [6].

### 2.2. The TJ and Its Regulators as Therapeutic Targets 

Barrier restoration and improvement are the main aim when targeting TJs and their proteins. Here, on the one hand, natural substances—which are already often used to treat pathological conditions in traditional medicine—are in focus; on the other hand, targeted modifications of proteins and peptides are used to induce TJ modulation.

Among the many studied cytokines that influence the barrier during inflammation, tumor necrosis factor-α (TNF) has been studied the most and is also linked to regulatory factors like myosin light chain kinase (MLCK) [7]: activated MLCK triggers the reorganization of the intestinal TJ via perijunctional actomyosin ring contraction, which further leads to TJ protein endocytosis and thus barrier changes. MLCK inhibition leads to toxic effects, as other essential enzymatic effects are inhibited too, but a small molecule—divertin—prevents only MLCK1 recruitment to the pathologic antibody-mediated rejection (PAMR), making it a potential therapeutic approach.

The effects of natural compounds are analyzed regarding their modes of action and signaling mechanisms beyond. For example, quercetin, a very common flavonoid, is known to improve the intestinal TJ barrier and also to reduce the occurrence of kidney stone formation by the reduction of ion and water reabsorption due to inducing PI3K/AKT-regulated claudin expression changes [8]. Curcumin, another polyphenol, shows protective effects in pathological conditions, which can be induced by infections with *Campylobacter jejuni*. This bacterial strain affects the TJ barrier via immune cell-mediated cytokine effects, which can be alleviated by curcumin [9].

Besides barrier enhancement and thus often protective effects, food compounds can also weaken or open the paracellular barrier. Caprate, a fatty acid, can reversibly remove claudin-5 and tricellulin from the TJ and, by this, modulate the intestinal barrier’s function. This also is observed in the immune-relevant Peyer’s patch follicle-associated epithelium, suggesting that caprate could trigger paracellular antigen uptake and might be connected to food allergy development [10]. However, the targeted opening of the TJ can be used to allow not only antigen uptake, as a more unwanted effect, but also enhanced drug uptake, which might be of special interest for the blood–brain barrier (BBB) or for treating tumors.

Numerous approaches have been taken to modify binding peptides originating from bacterial toxins. Such toxins can bind to specific TJ proteins; it is known that CPE can bind to members of the claudin family and that the *Clostridium perfringens* iota-toxin can bind to other TJ proteins such as the angulins. Peptide fragments that still possess binding capacities can be either used based on their direct binding to their original targets or can be mutated to gain binding specificity for other TJ proteins [3]. In detail, the mutation of the claudin-binding domain of CPE (cCPE) leads to its binding to the usually-not-targeted claudin-1, which is involved, for example, as a co-receptor for hepatitis C virus (HCV) infections or is a main component of the epidermal barrier limiting drug delivery. Such new interactions created by specifying the cCPE might thus allow its usage for therapeutic claudin-1 targeting or drug delivery [2].

### 2.3. Molecular Structure of TJ Networks and Proteins 

To achieve the specific modification of binding peptides and to understand the basic principles of TJ protein interaction and the formation of barriers and channels, knowledge of the molecular structure of TJ proteins is essential. Technical approaches to elucidate the structural features of TJs and the interaction of TJ proteins within the TJ network include super-resolution imaging, which resolves strand formation and appearance at levels already comparable to those achieved with freeze-fracture electron microscopy. Here, different aspects are in development, and technical challenges still have to be taken into account [11].

A higher resolution is achieved by crystal structure analyses, which, for a long time, were difficult for TJ proteins. It took a long time from the first published structures of JAM-a [12] and the cytosolic C-terminus of occludin [13] for the first complete claudin structure to be published [14]. In this Special Issue, an overview is given over the molecular structures of TJ proteins published to date, focusing also on structural motifs and the types of interactions between TJ proteins and their ligands involving these motifs [15]. The resolution of such structural features of TJ proteins may further be used to model interactions and to suggest the organization of these proteins to form TJ strands with dynamic barrier- and channel-forming properties [16]. The understanding of such interactions and motifs may furthermore allow the specification and development of new compounds targeting TJs. For claudin-5, the predominant claudin of the BBB, *in silico* modulations have been performed to understand how this claudin may be targeted and have also suggested interface formations, which, however, need to be confirmed by experimental studies [17].

## 3. Tight Junctions as Regulators of Cellular Processes 

### 3.1. TJ and Cell Polarity 

The interactions of TJ proteins with each other not only build paracellular barriers but also maintain the organization and separation of apically and basolaterally located membrane proteins and lipids via a fence function. Thus, they are essential for polarity and thereby the organization of epithelial functionality. Vice versa, polarity complex proteins influence TJ network formation and the resulting paracellular barrier. Polarity and the associated TJ barriers are also essential for transport processes, depending on solute gradients and/or electrical potentials. For the paracellular transport of phosphatidylcholine in the biliary tract, such negative potentials are essential and generated by cystic fibrosis transmembrane conductance regulator (CFTR) and the anion exchange protein 2 (AE2) [18].

The regulation of polarity, which is not only based on the TJ and polarity complex proteins but also involves the adherens junction (AJ), depends on several regulatory proteins. One of these is the adenosine monophosphate (AMP)-activated protein kinase (AMPK), which is well-known as a central regulator of energy metabolism. However, besides that, it is an upstream regulator of the AJ in response to junctional tension and affects TJ function and apicobasal cell polarization. Although still not completely understood, the functions and regulation of and via AMPK are reviewed in this Special Issue and may give further indications for AMPK as a potential target in barrier and polarity regulation [19].

Polarity complexes are also a target of pathogens, as their interruption also leads to barrier disturbance. Enteropathogenic *Escherichia coli* (EPEC) secrete the effector protein EspF, which leads to the disruption of the scaffold protein PAR (partitioning-defective) complex, instability of the actin cytoskeleton, and perturbation of the TJ via aPKCζ [20].

### 3.2. TJ and Cell Proliferation 

Closely connected to cell polarity is also the ability of cells to proliferate, and the crosstalk between both fundamental cellular processes is known. The control of epithelial and endothelial cell proliferation is not only linked to the adherens junction (AJ); the involvement of the TJ is increasingly being elucidated. TJ proteins may act as critical cell cycle modulators by binding to and regulating nuclear access of other factors. Furthermore, they may regulate transcription and proliferation by being located at cellular organelles instead of the apicolateral cell membranes. In this Special Issue, a review on TJs and cell proliferation explains the control of cell proliferation and differentiation by TJs, which is required for the formation and also maintenance of tissue barriers [21]. In this context, TJ-associated proteins that are not anchored to the membrane like the ZO proteins or cingulins seem to be especially important mediators, as they connect the cytoskeleton to the TJ and thereby have a huge impact on cytoarchitecture and regulation. One example of a protein involved as a key regulator in gene expression, cell proliferation, and cell size is ZO-2 [22].

### 3.3. Regulatory Functions of Tricellular TJ Proteins 

The tricellular tight junction (tTJ) is mainly involved in the regulation of paracellular macromolecule passage, and in tight epithelia, it also significantly contributes to ion permeability. Besides such classical functions of TJ proteins, tricellulin turned out to be able to directly regulate paracellular water transport in tight epithelial cells [23], and it was found that the tTJ is also of importance for cell and tissue differentiation. For example, during apoptosis, the tTJ is dissolved as a consequence of tricellulin fragmentation and the resulting instability of the tTJ [24]. In the differentiation of cells and tissues, the expression and localization of tTJ proteins is also of significance. For endometrial tissue, the menstrual cycle-dependent expression and localization of tricellulin and angulin-1 have been shown, and changes in these lead to dysfunctional barriers, promoting cancer progression and metastasis [25].

## 4. Organ- and Tissue-Specific Functions 

The expression profile of TJ proteins within different tissues and organs is very specific and does not only have general barrier or regulatory functionality for endothelia and epithelia. Within an organ, this profile is essential for the whole organ’s function, which can be very complex and thus easily and severely affected by small changes in the TJ protein composition or functionality of single proteins. To study the function and impact of single TJ proteins in such complexity, often, mouse models are generated that can provide information on human-associated disorders. Such models may be knock-outs, knock-downs, or knock-ins of the respective proteins [26], and the findings can be related to observed pathologies in patients. However, interspecific differences need to be taken in account, which, for example, can be observed in intestinal preservation injury, where progression at the tissue and molecular levels differs between rats, pigs, and humans [27].

From pathological conditions, conclusions are also drawn as to the actual physiological function within a complex organ or tissue. Some organs and the function of specific TJ proteins within are focus of this Special Issue.

### 4.1. Sensory Organs 

In the inner ear, for example, the maintenance of ion gradients and therefore strictly regulated barriers are necessary for hearing. Several loss-of-function mutations in TJ proteins have been linked to deafness. A novel variant of claudin-14, besides nine others, is linked to the phenotypic expansion of hereditary hearing loss, DFNB2 [28].

Another sensory organ is the eye, where, unlike for other epithelia, the apical surface of the retinal pigment epithelium (RPE) is in direct contact with neural tissue. It is involved in the daily phagocytosis of the effete tips of photoreceptor cells, and besides the well-studied transcellular trafficking of these, here, the role of TJs is still under investigation. However, the molecular composition of the TJ of the RPE is affected in several retinopathies, underlying an important function of this barrier [29].

### 4.2. Barriers of the Brain and Nerves 

The disturbance of the epithelial and endothelial barriers within the brain may be caused by different pathologies. One example is cerebral cavernous malformation (CCM), a disease characterized by mulberry-shaped clusters of dilated microvessels leading to seizures, headaches, and strokes from brain bleeding. A group of genes (CCM genes) that are involved in cellular signaling and barrier function may possess mutations leading to loss of function and thereby resulting in this pathology, but they may be also involved in others [30]. As the brain is an organ where epithelial, endothelial, and glial barriers are involved in functionality, high complexity in barrier formation and maintenance is essential. While the endothelial blood–brain barrier (BBB) and the epithelial blood–cerebrospinal fluid barrier (BCSFB) have been often studied, less is known about the epithelial and mesothelial arachnoid barrier and the glia limitans. Besides being physical barriers, they seem to also be of importance in immune compartmentation and immune surveillance [31].

Additionally, the nervous system possesses specialized barriers as, for example, the dorsal root ganglion (DRG) consists of neuron-rich and fiber-rich regions, and their putative epineurium/perineurium. The blood–DRG barrier (BDB) properties in these physiologically distinct regions are altered after nerve injury by changes in specific spatial TJ protein expression. For example, in the neuron-rich regions, claudin-5 was specifically downregulated, leading to the higher permeability of this barrier, while the other regions were not affected [32].

### 4.3. Gastrointestinal Organs 

In the liver, TJ proteins maintain tissue homeostasis and regulate paracellular diffusion between adherent hepatocytes or cholangiocytes at the blood–biliary barrier (BBIB). Non-junctional localization has regulatory cell functions such as those in differentiation, proliferation, and migration. In addition, TJ proteins are hepatocyte entry factors for the hepatitis C virus (HCV), and impaired TJ protein expression has been reported in several hepatobiliary diseases [33]. TJs are essential for the maintenance of negative potentials as driving forces for the paracellular transport of phosphatidylcholine in the biliary tract needed for mucus production [18].

In the intestine, in classic studies, barrier functions and transport have been investigated as the main functions of TJs. However, interactions with immune cells, mainly via their secreted cytokines in inflammatory processes, have been also described in a barrier-regulating context (see Section 2.1). The interaction of TJs and transcellular transporters do not only affect transport and typical barrier properties but also play a role in intestinal epithelial repair. TJ junction complex components are linked to ion channels and transporters that respond to loss of the homeostasis within the intestine during acute injury and finally induce TJ closure [34].

### 4.4. The Kidney 

Within the kidney, nephrons are the functional units regulating the re-uptake of nutrients, ions, and water as well as the excretion of unwanted substances and excess ions and water. Here, it is a pre-requisite that the different segments are well regulated in their function and that the TJ highly influences this. Claudins in the renal collecting duct differ in their expression and function [35] compared to those of the proximal segments. For example, in the distal regions, claudin-7 modulates salt homeostasis and WNK4 expression [36], and claudin-19 is finely adjusted in its function depending on the osmolality [37].

In the proximal tubule, claudin-12 has been shown to be important for permeability to calcium, as the proximal tubules of claudin-12 knock-out (KO) mice had different permeabilities. However, a decreased permeability to calcium in the proximal tubule was compensated by the downregulation of claudin-14 in the thick ascending limb of the Henle’s loop [38].

Claudin-10a is also expressed in the proximal tubules of the kidney but facilitates anion permeability. Defects in claudin-10a thus affect the proximal tubule, while claudin-10b is expressed in the thick ascending limb of the Henle’s loop but also in many other organs, such as the skin, salivary glands, sweat glands, brain, lung, and pancreas; pathological changes can be observed in these as well [39]. Such effects can be very different in the specific organs, which is also known for other claudins. Claudin-2, which is another channel-forming TJ protein, not only regulates paracellular cation and water transport within organs such as the proximal tubules, small intestine, liver, gall bladder, and pancreas but is also involved in proliferation, migration, and cell fate determination by the alteration of key signaling pathways [40].

## 5. Tight Junctions Sense and React to Environmental Conditions 

Environmental factors also belong to influences that affect barrier integrity and thereby lead to pathologies. Ultraviolet (UV) radiation, like UVB, and oxidative stress in skin epithelial cells (HaCaT) lead to the dephosphorylation of claudin-1, which then is removed from TJs, leading to their disruption. This effect can be rescued by an extract of Brazilian green propolis [41].

In euryhaline fish, the change between fresh and saltwater conditions leads to adaptive changes to allow the handling of the imbibed seawater [42]. In the fish intestine, transcellular aquaporin-dependent water transport is downregulated, and paracellular transport via the two paralogs of claudin-15 present in fish, cldn15a and cldn15b, plays a major role.

The environment can be comprised of the outer environmental condition, but also the microenvironment in a tissue or a cell. Already, under physiological conditions, the TJ forming the paracellular barrier is dynamically regulated. Microenvironmental factors change the tissue’s properties and lead to adaptation to the new conditions. Such factors may be osmolality changes, which can be huge and are of significance, especially in the kidney. In the inner medullary collecting duct, claudin-19 expression and localization in TJs or vesicles depend on osmolality, which leads to transformation from tight to leaky epithelia and vice versa, due to changes in ion transport and ion selectivity [37]. Osmolality and hydrostatic pressure furthermore affect not only transepithelial transport but also cell proliferation and the cytoskeletal architecture. Changes in these parameters may be sensed by TJ proteins and also play a role in carcinogenesis [43].

Aging, in addition, results in changes in microenvironmental conditions. For example, age-dependent changes in the immune system can be linked to pathologies affecting the TJs of the blood–brain barrier (BBB), altering the microvasculature and, by this, possibly impairing cognitive skills and abilities, which can be observed in several age-related disorders like Alzheimer’s or Parkinson’s disease [44]. The TJ attains increasing focus, as barrier dysfunction and microenvironmental changes appear to be common denominators in neurological disorders.

## 6. TJ Proteins and Cancer 

Facing the fact that tumor and cancer development is usually based on various disturbed cell processes, it is obvious that many different factors can be involved. Besides the clearly regulatory factors of proliferation, the TJ and its proteins are also affected. Besides their role in the typically connected barrier integrity and paracellular transport regulation, TJ proteins are involved in many other aspects. This makes TJ proteins also important for such processes that are strictly regulated but out of control in cancer. Many types of cancer exhibit changes in the expression of TJ proteins. Some of them are also used as markers for cancer subtypes. 

In general, it is assumed that the barrier impairment and loss of polarity due to TJ dysregulation are responsible for tumor progression and metastasis. However, the role of distinct TJ proteins within tumorigeneses is less understood, and some claudins can be upregulated in one kind of cancer while being downregulated in another. Nevertheless, claudins are emerging targets for therapeutic approaches and the elucidation of cancer malignancy, as they have been shown to be causally linked to mesenchymal transition [45]. Especially, on the example of claudin-1, it becomes clear that it can be involved in different ways in cancer development and that the generation of therapeutics relies on further investigations that explore the involvement of the respective claudins in the different types of cancer [46].

## 7. Conclusions 

Knowledge about the TJ and its proteins has evolved from the early findings of its role in sealing the paracellular cleft of epithelia and endothelia, establishing barriers, and maintaining gradients towards a more differentiated view that these barriers are selective in their properties—as some TJ proteins form paracellular channels through the bTJ—and that different paracellular pathways exist for small ions, small uncharged solutes, water, and macromolecular solutes. In addition, the paracellular pathways regulated by the TJ are of a dynamic nature and are well-regulated under physiological conditions but are also affected under pathological states.

Besides such properties that influence the formation and regulation of paracellular barriers, an increasing number of TJ proteins involved in various cellular processes and regulations are identified, indicating the diverse and dynamic functionality of these proteins. This Special Issue gives an overview of several of those mechanisms besides the well-established barrier function. The different aspects, covered by a total of 43 articles, demonstrate that the field of TJ research is about to explode.

## Abbreviations

AQP	Aquaporin
CPE	*Clostridium perfringens* enterotoxin
KD	Knock-down
KO	Knock-out
SGLT	Sodium-glucose-linked transporter
TAMP	Tight junction-associated MARVEL protein
TER	Transepithelial resistance
TJ	Tight junction
bTJ	Bicellular tight junction
tTJ	Tricellular tight junction
TM	Transmembrane
TNF-α	Tumor necrosis factor-α

## References

[1] Hering N.A., Fromm A., Bucker R., Gorkiewicz G., Zechner E., Hogenauer C., Fromm M., Schulzke J.D., Troeger H. (2019). Tilivalline- and tilimycin-independent effects of klebsiella oxytoca on tight junction-mediated intestinal barrier impairment. Int. J. Mol. Sci..

[2] Beier L.S., Rossa J., Woodhouse S., Bergmann S., Kramer H.B., Protze J., Eichner M., Piontek A., Vidal Y.S.S., Brandner J.M. (2019). Use of modified clostridium perfringens enterotoxin fragments for claudin targeting in liver and skin cells. Int. J. Mol. Sci..

[3] Hashimoto Y., Tachibana K., Krug S.M., Kunisawa J., Fromm M., Kondoh M. (2019). Potential for tight junction protein-directed drug development using claudin binders and angubindin-1. Int. J. Mol. Sci..

[4] Schmidt H., Braubach P., Schilpp C., Lochbaum R., Neuland K., Thompson K., Jonigk D., Frick M., Dietl P., Wittekindt O.H. (2019). Il-13 impairs tight junctions in airway epithelia. Int. J. Mol. Sci..

[5] Brewer M.G., Yoshida T., Kuo F.I., Fridy S., Beck L.A., De Benedetto A. (2019). Antagonistic effects of il-4 on il-17a-mediated enhancement of epidermal tight junction function. Int. J. Mol. Sci..

[6] Delbue D., Cardoso-Silva D., Branchi F., Itzlinger A., Letizia M., Siegmund B., Schumann M. (2019). Celiac disease monocytes induce a barrier defect in intestinal epithelial cells. Int. J. Mol. Sci..

[7] He W.-Q., Wang J., Sheng J.-Y., Zha J.-M., Graham W.V., Turner J.R. (2020). Contributions of myosin light chain kinase to regulation of epithelial paracellular permeability and mucosal homeostasis. Int. J. Mol. Sci..

[8] Gamero-Estevez E., Andonian S., Jean-Claude B., Gupta I., Ryan A.K. (2019). Temporal effects of quercetin on tight junction barrier properties and claudin expression and localization in mdck ii cells. Int. J. Mol. Sci..

[9] Lobo de Sa F.D., Butkevych E., Nattramilarasu P.K., Fromm A., Mousavi S., Moos V., Golz J.C., Stingl K., Kittler S., Seinige D. (2019). Curcumin mitigates immune-induced epithelial barrier dysfunction by campylobacter jejuni. Int. J. Mol. Sci..

[10] Radloff J., Cornelius V., Markov A.G., Amasheh S. (2019). Caprate modulates intestinal barrier function in porcine peyer’s patch follicle-associated epithelium. Int. J. Mol. Sci..

[11] Gonschior H., Haucke V., Lehmann M. (2020). Super-resolution imaging of tight and adherens junctions: Challenges and open questions. Int. J. Mol. Sci..

[12] Kostrewa D., Brockhaus M., D’Arcy A., Dale G.E., Nelboeck P., Schmid G., Mueller F., Bazzoni G., Dejana E., Bartfai T. (2001). X-ray structure of junctional adhesion molecule: Structural basis for homophilic adhesion via a novel dimerization motif. Embo J..

[13] Li Y., Fanning A.S., Anderson J.M., Lavie A. (2005). Structure of the conserved cytoplasmic c-terminal domain of occludin: Identification of the zo-1 binding surface. J. Mol. Biol..

[14] Suzuki H., Nishizawa T., Tani K., Yamazaki Y., Tamura A., Ishitani R., Dohmae N., Tsukita S., Nureki O., Fujiyoshi Y. (2014). Crystal structure of a claudin provides insight into the architecture of tight junctions. Science.

[15] Heinemann U., Schuetz A. (2019). Structural features of tight-junction proteins. Int. J. Mol. Sci..

[16] Fuladi S., Jannat R.-W., Shen L., Weber C.R., Khalili-Araghi F. (2020). Computational modeling of claudin structure and function. Int. J. Mol. Sci..

[17] Rajagopal N., Irudayanathan F.J., Nangia S. (2019). Computational nanoscopy of tight junctions at the blood-brain barrier interface. Int. J. Mol. Sci..

[18] Stremmel W., Staffer S., Weiskirchen R. (2019). Phosphatidylcholine passes by paracellular transport to the apical side of the polarized biliary tumor cell line mz-cha-1. Int. J. Mol. Sci..

[19] Tsukita K., Yano T., Tamura A., Tsukita S. (2019). Reciprocal association between the apical junctional complex and ampk: A promising therapeutic target for epithelial/endothelial barrier function?. Int. J. Mol. Sci..

[20] Tapia R., Kralicek S.E., Hecht G.A. (2020). Enteropathogenic escherichia coli (epec) recruitment of par polarity protein atypical pkcζ to pedestals and cell–cell contacts precedes disruption of tight junctions in intestinal epithelial cells. Int. J. Mol. Sci..

[21] Díaz-Coránguez M., Liu X., Antonetti D.A. (2019). Tight junctions in cell proliferation. Int. J. Mol. Sci..

[22] González-Mariscal L., Gallego-Gutiérrez H., González-González L., Hernández-Guzmán C. (2019). Zo-2 is a master regulator of gene expression, cell proliferation, cytoarchitecture, and cell size. Int. J. Mol. Sci..

[23] Ayala-Torres C., Krug S.M., Schulzke J.D., Rosenthal R., Fromm M. (2019). Tricellulin effect on paracellular water transport. Int. J. Mol. Sci..

[24] Janke S., Mittag S., Reiche J., Huber O. (2019). Apoptotic fragmentation of tricellulin. Int. J. Mol. Sci..

[25] Kohno T., Konno T., Kojima T. (2019). Role of tricellular tight junction protein lipolysis-stimulated lipoprotein receptor (lsr) in cancer cells. Int. J. Mol. Sci..

[26] Seker M., Fernández-Rodríguez C., Martínez-Cruz L.A., Müller D. (2019). Mouse models of human claudin-associated disorders: Benefits and limitations. Int. J. Mol. Sci..

[27] Søfteland J.M., Casselbrant A., Biglarnia A.-R., Linders J., Hellström M., Pesce A., Padma A.M., Jiga L.P., Hoinoiu B., Ionac M. (2019). Intestinal preservation injury: A comparison between rat, porcine and human intestines. Int. J. Mol. Sci..

[28] Kitano T., Kitajiri S.-i., Nishio S.-y., Usami S.-i. (2019). Detailed clinical features of deafness caused by a claudin-14 variant. Int. J. Mol. Sci..

[29] Naylor A., Hopkins A., Hudson N., Campbell M. (2019). Tight junctions of the outer blood retina barrier. Int. J. Mol. Sci..

[30] Wei S., Li Y., Polster S.P., Weber C.R., Awad I.A., Shen L. (2020). Cerebral cavernous malformation proteins in barrier maintenance and regulation. Int. J. Mol. Sci..

[31] Castro Dias M., Mapunda J.A., Vladymyrov M., Engelhardt B. (2019). Structure and junctional complexes of endothelial, epithelial and glial brain barriers. Int. J. Mol. Sci..

[32] Lux T.J., Hu X., Ben-Kraiem A., Blum R., Chen J.T.-C., Rittner H.L. (2019). Regional differences in tight junction protein expression in the blood–drg barrier and their alterations after nerve traumatic injury in rats. Int. J. Mol. Sci..

[33] Roehlen N., Roca Suarez A.A., El Saghire H., Saviano A., Schuster C., Lupberger J., Baumert T.F. (2020). Tight junction proteins and the biology of hepatobiliary disease. Int. J. Mol. Sci..

[34] Slifer Z.M., Blikslager A.T. (2020). The integral role of tight junction proteins in the repair of injured intestinal epithelium. Int. J. Mol. Sci..

[35] Leiz J., Schmidt-Ott K.M. (2019). Claudins in the renal collecting duct. Int. J. Mol. Sci..

[36] Fan J., Tatum R., Hoggard J., Chen Y.-H. (2019). Claudin-7 modulates cl− and na+ homeostasis and wnk4 expression in renal collecting duct cells. Int. J. Mol. Sci..

[37] Ziemens A., Sonntag S.R., Wulfmeyer V.C., Edemir B., Bleich M., Himmerkus N. (2019). Claudin 19 is regulated by extracellular osmolality in rat kidney inner medullary collecting duct cells. Int. J. Mol. Sci..

[38] Plain A., Pan W., O’Neill D., Ure M., Beggs M.R., Farhan M., Dimke H., Cordat E., Alexander R.T. (2020). Claudin-12 knockout mice demonstrate reduced proximal tubule calcium permeability. Int. J. Mol. Sci..

[39] Milatz S. (2019). A novel claudinopathy based on claudin-10 mutations. Int. J. Mol. Sci..

[40] Venugopal S., Anwer S., Szászi K. (2019). Claudin-2: Roles beyond permeability functions. Int. J. Mol. Sci..

[41] Marunaka K., Kobayashi M., Shu S., Matsunaga T., Ikari A. (2019). Brazilian green propolis rescues oxidative stress-induced mislocalization of claudin-1 in human keratinocyte-derived hacat cells. Int. J. Mol. Sci..

[42] Tipsmark C.K., Nielsen A.M., Bossus M.C., Ellis L.V., Baun C., Andersen T.L., Dreier J., Brewer J.R., Madsen S.S. (2020). Drinking and water handling in the medaka intestine: A possible role of claudin-15 in paracellular absorption?. Int. J. Mol. Sci..

[43] Tokuda S., Yu A.S.L. (2019). Regulation of epithelial cell functions by the osmolality and hydrostatic pressure gradients: A possible role of the tight junction as a sensor. Int. J. Mol. Sci..

[44] Costea L., Meszaros A., Bauer H., Bauer H.C., Traweger A., Wilhelm I., Farkas A.E., Krizbai I.A. (2019). The blood-brain barrier and its intercellular junctions in age-related brain disorders. Int. J. Mol. Sci..

[45] Gowrikumar S., Singh A.B., Dhawan P. (2019). Role of claudin proteins in regulating cancer stem cells and chemoresistance-potential implication in disease prognosis and therapy. Int. J. Mol. Sci..

[46] Bhat A.A., Syed N., Therachiyil L., Nisar S., Hashem S., Macha M.A., Yadav S.K., Krishnankutty R., Muralitharan S., Al-Naemi H. (2020). Claudin-1, a double-edged sword in cancer. Int. J. Mol. Sci..

